# Transurethral marking incision of the bladder neck: a helpful technique in robot-assisted laparoscopic radical prostatectomy involving post-transurethral resection of the prostate and cancers protruding into the bladder neck

**DOI:** 10.1186/1471-2490-13-40

**Published:** 2013-08-17

**Authors:** Satoshi Kurokawa, Keiichi Tozawa, Yukihiro Umemoto, Takahiro Yasui, Kentaro Mizuno, Atsushi Okada, Noriyasu Kawai, Yutaro Hayashi, Kenjiro Kohri

**Affiliations:** 1 Department of Urology, Nagoya Tokushukai General Hospital, Kasugai, Japan; 2 Department of Nephro-urology, Nagoya City University Graduate School of Medical Sciences, 1, Kawasumi, Mizuho-cho, Mizuho-ku, 467-8601 Nagoya, Japan

**Keywords:** Bladder neck, Prostate cancer, Robot-assisted laparoscopic radical prostatectomy, Transurethral incision, Transurethral resection of the prostate

## Abstract

**Background:**

Bladder neck transection is one of the most difficult procedures for robot-assisted laparoscopic radical prostatectomy (RALP), particularly in patients who have undergone previous transurethral resection of the prostate (TUR-P), and in those with large median lobes or prostate cancer protruding into the bladder neck. To ensure negative surgical margins and safely preserve the ureteral orifices during bladder neck transection, we propose the use of the transurethral resectoscope for making the incision in the bladder neck before initiating RALP. Thus, we developed a technique for bladder neck transection to facilitate this operation in such patients.

**Case presentation:**

Two Japanese men, aged 61 and 63 years, who were diagnosed with prostate cancer, received a transurethral marking incision of the bladder neck before starting RALP; prostate cancer developed in one patient after TUR-P and the other patient had cancer protruding into the bladder neck. A transurethral resectoscope was used to closely observe the ureteral orifices and bladder necks; the bladder necks were marked to indicate the depth from the mucosa to the muscular layer. During the RALP, the bladder necks were dissected to indicate the depth of the marking incision. The surgical margins were negative and perioperative complications did not occur. The Foley catheters were removed on postoperative day 6, according to the usual protocol. No urinary leakage from the anastomosis sites was observed.

**Conclusion:**

This technique, involving the use of an ordinary transurethral resectoscope, may be an easy procedure to ensure negative surgical margins, safely preserve the ureteral orifices, avoid increasing the bladder neck diameter, and achieve a good quality vesicourethral anastomosis that prevents the risk of suture-related tissue tears.

## Background

Bladder neck transection is one of the most difficult procedures associated with robot-assisted laparoscopic radical prostatectomy (RALP), particularly in patients who have undergone previous transurethral resection of the prostate (TUR-P), and in those with large median lobes or prostate cancer protruding into the bladder neck. Among such patients, the rates of positive surgical margins and operative complications, such as ureteral orifice injury or obstruction, urinary leakage, or bladder neck contracture, are approximately twice as high as in other patients [1-4].

Certain techniques have been proposed for preserving the ureteral orifices during RALP procedures, including their enhanced identification in the robotic magnified view by the administration of intravenous furosemide and indigo carmine, and the insertion of ureteral stents [5,6]. During such techniques, the surgeon has to sufficiently enlarge the diameter of the bladder neck to clearly observe the bilateral ureteral orifices, and the magnified view from the surgeon’s console may not be suitable for observing the ureteral orifices. Moreover the transurethral cystoscope is more suitable than the da Vinci’s laparoscope for observing ureteral orifices. In the present study, we propose the use of the TUR system to closely observe the ureteral orifices and for making a transurethral marking incision in the bladder neck before initiating RALP.

## Case presentation

Case 1: A 61-year-old man presented with dysuria; his serum prostate-specific antigen (PSA) level was 2.2 ng/mL. TUR-P was performed, and an adenocarcinoma (Gleason score 4 + 3) was incidentally detected in 8% of the resected tissue. He was scheduled to undergo radical prostatectomy at 3 months after TUR-P described as a recommended duration in recent reports [7-9].

**Figure 1:**
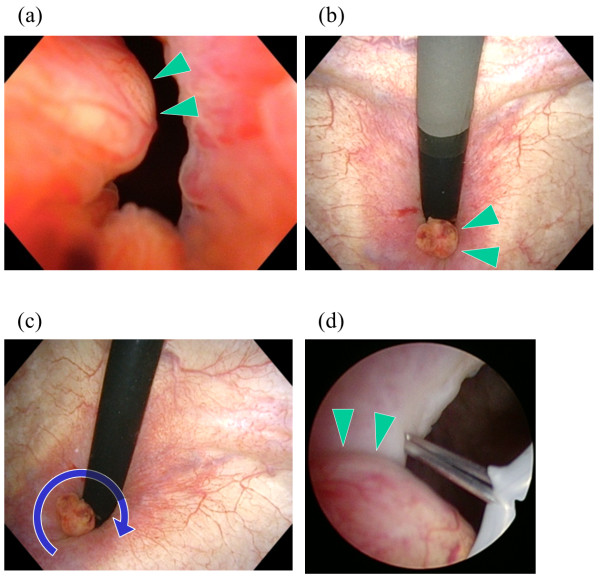
**Urethrocystoscopic view in case 2. (a**, **b)** Appearance of the prostate cancer protruding into the bladder neck from the prostatic urethra, during flexible urethrocystoscopy. **(c**, **d)** Transurethral incision of the bladder neck with the knife-type electrode of the transurethral resection system.Arrowheads indicate the prostate ductal adenocarcinoma protruding into the bladder neck. Arrows indicate the transurethral incision of the bladder neck.

Case 2: A 63-year-old man presented with gross haematuria and pollakisuria; his serum PSA level was 30.0 ng/mL. Flexible urethrocystoscopy indicated the presence of a solid tumour, approximately 1 cm in size, protruding into the bladder neck from the prostatic urethra (Figure 1a,b). Contrast-enhanced magnetic resonance imaging also indicated the presence of tumour enhancement at the bladder neck, which may have represented a urothelial cancer invading the prostate or a prostate cancer projecting to the bladder neck. A transrectal tumour biopsy, performed under ultrasonographic guidance, indicated a histopathological diagnosis of ductal adenocarcinoma of the prostate. He was diagnosed as having prostate cancer protruding into the bladder neck. Contrast-enhanced computed tomography from the chest to the pelvis and a bone scan demonstrated the absence of metastases, and the patient was scheduled to undergo a radical prostatectomy.

**Figure 2:**
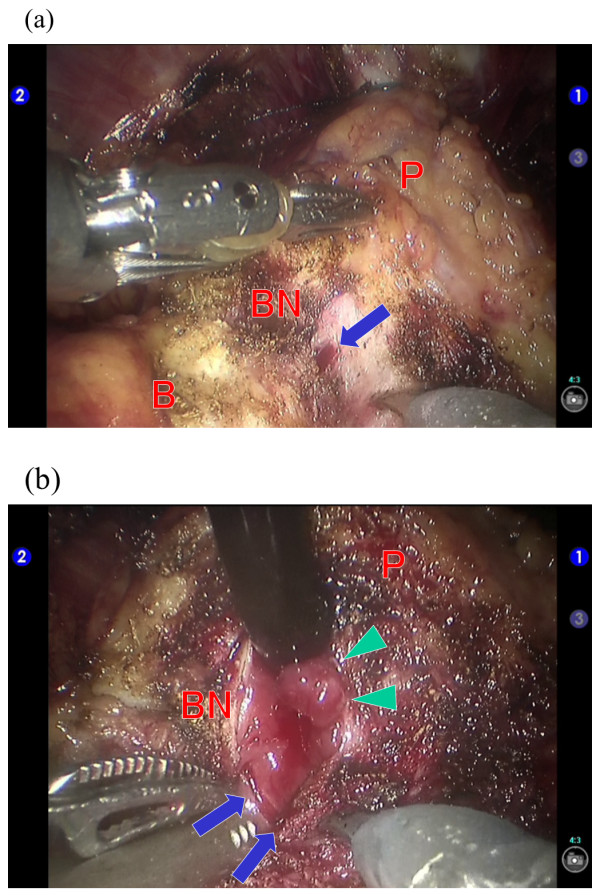
**Robotic view in case 2. (a)** The bladder neck transection reached the depth of the transurethral marking incision. **(b)** The bladder muscle was incised along the line of transurethral marking incision. P, prostate; B, bladder; BN, bladder neck. Arrowheads indicate the prostate ductal adenocarcinoma protruding into the bladder neck. Arrows indicate the transurethral incision of the bladder neck.

The operative procedures involved making transurethral marking incisions in the bladder necks using the TUR system’s knife-type electrode (30° optic, monopolar, Olympus, Tokyo, Japan). Before initiating RALP, each patient was placed in a flat lithotomy position, and the distance to the ureteral orifices was measured; the prostate cancer was found to protrude into the bladder neck in case 2 (Figure 1c,d). All the marking incisions, extending from the mucosa to the muscular layer, were made within approximately 10 minutes. After the transurethral incisions were made, the operating bed was tilted to the Trendelenburg position and the RALP was performed by using a 4-arm da Vinci S unit (Intuitive Surgical, Sunnyvale, CA, USA). During the RALP procedure, we carefully continued with the bladder neck transection, by cutting each layer of the bladder detrusor muscle to the depth of the transurethral marking incision (Figure 2a). Finally, the bladder muscle was incised along the line of the transurethral marking incision to complete the transection (Figure 2b). The time to complete the robotic aspects of the surgeries were 112 min (case 1) and 148 min (case 2), which did not markedly differ from the average robotic time for ordinary RALP, in our experience of 301 cases (average, 157 minutes).

**Figure 3:**
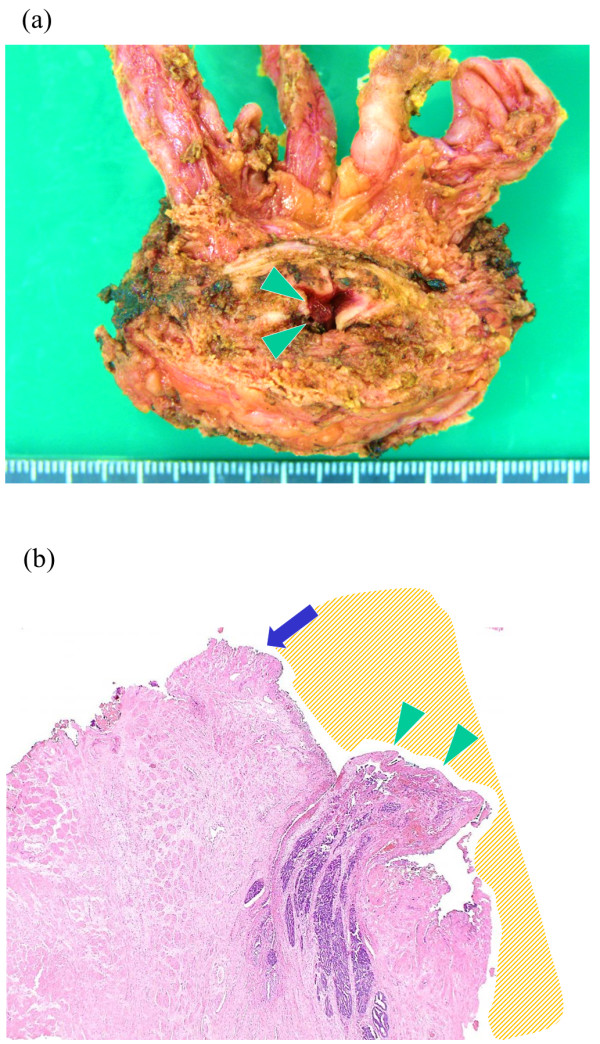
**Surgical specimen and pathological findings in case 2. (a)** Gross appearance of the excised prostate, with protruding ductal adenocarcinoma. **(b)** Haematoxylin-eosin stain (original magnification, ×20). The shaded area represents the urethral lumen. The prostate ductal adenocarcinoma protruding into the bladder neck is located at a distance from the edge of transurethral marking incision. Arrowheads indicate the prostate ductal adenocarcinoma protruding into the bladder neck. Arrows indicate the transurethral incision of the bladder neck.

In both patients, the surgical margins were negative, as clearly noted on microscopic examination of a specimen from case 2 (Figure 3a,b), and no perioperative complications developed. After performing retrograde and voiding cystourethrography to confirm that no urine leakage was present at the anastomosis site, the Foley catheter was removed from each patient on postoperative day 6, according to the usual protocol. A few days after the removal of the Foley catheters, the patients were discharged from the hospital. Three months after the RALP, the serum PSA levels of both patients were < 0.2 ng/mL, and they had acceptable urinary continence, with the use of one safety pad per day.

Both the patients provided informed consent, and the Institutional Review Board of Nagoya Tokushukai General Hospital approved the publication of these results (approval numbaer 2011-3).

## Conclusions

In our experience, these 2 cases were the 69^th^ and 93^rd^ cases. At the early stage in the learning curve of robotic surgery, RALP would have been difficult to perform in both of these cases, including a case of cancer developing after TUR-P and a case of prostate cancer protruding into the bladder neck. Intuitive Surgical, manufacturers of the da Vinci system, indicates that any surgeon who has completed 20 robotic operations is a potential proctor. Recent reports indicate that the learning curve of functional and oncological outcomes begins to plateau at approximately 50–100 cases [10-12]. The described technique may be performed easily and safely as the surgeon’s experience nears the plateau of the learning curve. Before performing RALP, we gained experience with 579 cases of laparoscopic radical prostatectomy (LRP) [13]. We experienced a smooth transition from LRP to RALP with a low complication rate, including a 0.3% rate of blood transfusions, no visceral injuries, and no conversions to open surgery as described in recent reports [14,15]. Among the 579 cases of LRP, we have performed transurethral marking incisions of the bladder neck, preoperative insertion of ureteral stents, and both these procedures simultaneously in cases who underwent prior TUR-P. However, transurethral incision of the bladder neck is a safe and satisfactory procedure, compared with ureteral stenting alone or both procedures performed simultaneously.

The use of a transurethral marking incision in the bladder neck was shown to facilitate bladder neck transections during RALP, especially when the anatomy of the bladder neck is altered. This procedure can be easily and quickly performed using an ordinary TUR system. Thus, we believe that this technique may be useful for ensuring negative surgical margins, safely preserving the ureteral orifices, avoiding increases in bladder neck diameter, and achieving a good quality vesicourethral anastomosis that prevents the risk of suture-related tissue tears.

## Consent

Written informed consent was obtained from the patients for publication of this case report and accompanying images. A copy of the written consent is available for review by the Editor of this journal.

## Abbreviations

PSA: Prostate-specific antigen; LRP: Laparoscopic radical prostatectomy; RALP: Robot-assisted laparoscopic radical prostatectomy; TUR: Transurethral resection; TUR-P: Transurethral resection of the prostate.

## Competing interests

The authors declare that they have no competing interests.

## Authors’ contributions

SK treated the patients and drafted the manuscript. KT participated in the discussions on the study concept and treated the patients. YU, TY, KM, AO, NK, YH, and KK participated in the discussions on the study concept. All authors read and approved the final manuscript.

## Pre-publication history

The pre-publication history for this paper can be accessed here:

http://www.biomedcentral.com/1471-2490/13/40/prepub
